# C-Peptide and Atherogenesis: C-Peptide as a Mediator of Lesion Development in Patients with Type 2 Diabetes Mellitus?

**DOI:** 10.1155/2008/385108

**Published:** 2008-04-01

**Authors:** Nikolaus Marx, Daniel Walcher

**Affiliations:** Department of Internal Medicine II—Cardiology, University of Ulm, Ulm 89073, Germany

## Abstract

Patients with insulin resistance and early type 2 diabetes exhibit an increased propensity to develop a diffuse and extensive pattern of arteriosclerosis. Typically, these patients show increased levels of C-peptide and over the last years various groups examined the effect of C-peptide in vascular cells as well as its potential role in lesion development. While some studies demonstrated beneficial effects of C-peptide, for example, by showing an inhibition of smooth muscle cell proliferation, others suggested proatherogenic mechanisms in patients with type 2 diabetes. Among them, C-peptide may facilitate the recruitment of inflammatory cells into early lesions and promote lesion progression by inducing smooth muscle cell proliferation. The following review will summarize the effects of C-peptide in vascular cells and discuss the potential role of C-peptide in atherogenesis in patients with type 2 diabetes.

## 1. INTRODUCTION

Patients with diabetes and insulin resistance exhibit an increased propensity to develop arteriosclerosis with its sequelae acute myocardial infarction and stroke [1]. Due to peripheral insulin resistance, these patients temporarily demonstrate elevated levels of the proinsulin cleavage product C-peptide. For a long time, C-peptide has been considered to be biological inert until recent work in kidney cells suggest that C-peptide can activate intracellular signaling pathways such as NA-K-ATPase [2, 3]. In addition, experimental data have shown activation of MAP kinase in fibroblasts and lung endothelial cells as well as activation of protein kinase C and PI3-kinase through C-peptide [4, 5]. These data suggested that C-peptide may be biologically active. Moreover, various groups demonstrated that C-peptide administration in patients with type 1 diabetes mellitus ameliorates diabetes-induced renal and nerve dysfunction [6, 7]. Wallerath et al. demonstrated that C-peptide stimulates the release of NO in endothelial cells in vitro and that this effect is mediated by an induction of Ca^2+^ influx into the cells [8]. Such mechanism may also explain some of the beneficial effects of C-peptide in type 1 diabetes.

In addition, recent work addressed effects of C-peptide in vascular cells; the following review will focus on these effects and discuss the potential relevance for atherogenesis in patients with type 2 diabetes.

## 2. ATHEROGENESIS

According to our current understanding, atherogenesis is an inflammatory process in the vessel wall with different phases and stages [9]. The early phase, before any appearance of microscopic or macroscopic changes, is characterized by endothelial dysfunction. Under the influence of cardiovascular risk factors, the endothelium looses its integrity and becomes permeable for plasma compounds like low-density lipoprotein (LDL) which can enter the vessel wall and deposit in the subendothelial space. In addition, during endothelial dysfunction the endothelium releases cytokines and chemotactic proteins and expresses adhesion molecules on the cell surface. Such mechanisms facilitate the recruitment of monocytes and CD4^+^ lymphocytes, important inflammatory cells during lesion development [10]. Once these cells have entered the vessel wall, monocytes differentiate to macrophages and express scavenger receptors on their surface to promote the uptake of oxidized LDL. These lipid-loaden cells then become foam cells, the classical cellular substrate of fatty-streaks. Foam cells release various kinds of proinflammatory and prothrombotic mediators and play a critical role in plaque progression. CD4^+^ lymphocytes are also attracted by chemokines and enter the vessel wall as naïve TH0 cells. In the subendothelial space, these cells then encounter antigens like oxidized LDL and differentiate towards TH1 cells which release proinflammatory cytokines such as IFN*γ*, TNF*α*, and IL-2. Some of these cytokines then enhance endothelial expression and release of T-cell specific chemokines, creating a vicious cycle of cell activation and cell recruitment [11]. In addition, these TH1 cytokines activate other cells in the vessel wall like macrophages and smooth muscle cells (SMCs), thus orchestrating the inflammatory response in the vessel wall. With increased recruitment of these inflammatory cells fatty streaks develop and SMCs from the media start to proliferate and migrate into the intima. As lesion formation progresses, advanced and potentially complicated lesions are formed. These lesions may lead to a progressive narrowing of the vessel wall thus potentially creating stable angina if located in the coronary artery. Alternatively, these plaques may become vulnerable and upon rupture can cause an acute coronary syndrome [12].

## 3. C-PEPTIDE DEPOSITION IN EARLY ARTERIOSCLEROTIC LESIONS

Since endothelial dysfunction with increased permeability occurs in patients with insulin resistance and early type 2 diabetes, a group of patients with temporarily high C-peptide serum levels, it has been hypothesized that C-peptide might deposit in the vessel wall in these patients in early atherogenesis. Immunohistochemical analyses of early arteriosclerotic lesions of patients with diabetes from the PDAY study (pathobiological determinants of atherosclerosis in youth) revealed C-peptide deposition mainly in the subendothelial space and the intima. Some of the diabetic subjects also exhibited C-peptide deposition in the media. In contrast, only very little C-peptide deposition has been found in early arteriosclerotic lesions of nondiabetic subjects. Computer-assisted analyses revealed significantly higher C-peptide deposition in lesions from diabetic individuals compared to lesions of age, sex, and risk factor matched nondiabetic subjects [13]. Interestingly, no deposition of insulin or proinsulin was detectable in diabetic or nondiabetic subjects. Staining of parallel sections as well as immunofluorescence techniques demonstrated colocalisation of C-peptide with intimal monocyte/macrophages and CD4^+^ lymphocytes in some of the diabetic individuals [14]. In the studies cited above, C-peptide deposition has been found in 100% of the 21 diabetic individuals examined, while monocyte infiltration was only present in 77%, and CD4^+^ lymphocyte infiltration only in 57%. These data suggested that C-peptide deposition may precede monocyte and T-cell migration into the vessel wall. Based on this observation, the hypothesis was raised that C-peptide may deposit in the vessel wall during early atherogenesis and then—through chemotactic effects—promote the recruitment of monocytes and CD4^+^ lymphocytes. Still, it remains unclear to what extent other peptides may also deposit in the subendothelial space and exhibit similar effects.

## 4. CHEMOTACTIC ACTIVITY OF C-PEPTIDE TOWARDS MONOCYTES AND CD4^+^ LYMPHOCYTES

In vitro migration assays, employing a modified Boyden chamber, revealed that C-peptide induces the migration of both monocytes and CD4^+^ lymphocytes in a concentration- dependent manner. Interestingly, insulin did not have such an effect. The chemotactic effects of C-peptide on these cells were within the range of very well-established chemokines like MCP-1 and RANTES. In addition, checkerboard analyses showed that C-peptide induces chemotaxis rather than chemokinesis [13, 14]. Interestingly, C-peptide does not exhibit any chemotactic activity towards neutrophils, cells which are not present in arteriosclerotic lesions.

Inhibition migration assays as well as western blot analyses and activity assays demonstrated that C-peptide mediates its chemotactic activity through an as of yet unidentified pertussis toxin sensitive G-protein coupled receptor with subsequent downstream activation of PI3-kinase *γ*.

**Figure 1:**
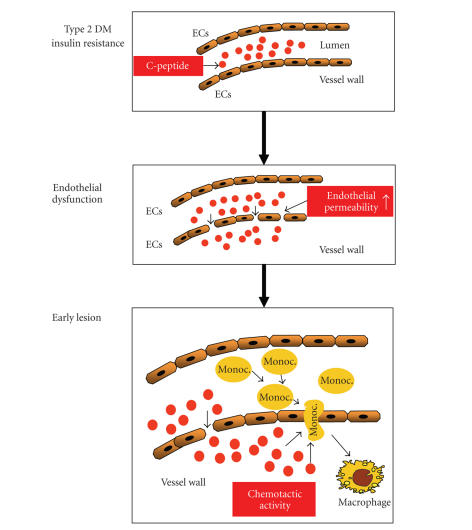
Potential role of C-peptide in early atherogenesis in patients with insulin resistance and early type 2 diabetes mellitus. During endothelial dysfunction with increased endothelial permeability, C-peptide could deposit in the intima and through its chemotactic activity on monocytes and CD4^+^ lymphocytes facilitate the recruitment of these inflammatory cells into the vessel wall.

In summary, these data suggest that—during endothelial dysfunction with increased endothelial permeability—C-peptide may deposit in the arterial intima in patients with insulin resistance and early type 2 diabetes mellitus and subsequently attract monocytes and CD4^+^ lymphocytes into the vessel wall (Figure 1). Such mechanisms may promote lesion development and potentially explain why patients with diabetes develop such a diffuse and extensive pattern of arteriosclerosis at a very early time point.

In addition to these proatherogenic effects, interesting data in monocyte-like THP1 cells showed that C-peptide increases the expression of CD36, an important scavenger receptor for the macrophage uptake of oxidized LDL in arteriosclerotic lesions [15]. These data suggest that C-peptide—in addition to its chemotactic effects on monocytes—may also promote the differentiation of monocyte/macrophages towards foam cells, thus representing another potential proatherogenic effect of C-peptide.

## 5. C-PEPTIDE AND SMOOTH MUSCLE CELL PROLIFERATION

Since C-peptide also colocalized with SMCs in the media of early arteriosclerotic lesions in some diabetic subjects, it has been suggested that C-peptide could also exhibit biological activity in these cells [16]. SMCs play a crucial role in the development of arteriosclerotic lesions and the formation of fatty streaks. In addition, SMCs are of critical importance in the formation of restenosis after coronary intervention: after vascular injury these cells start to proliferate and then migrate into the developing neointima, becoming the major cellular substrate of the restenotic tissue [17]. These mechanisms seem to be accelerated in patients with diabetes and insulin resistance, thus contributing to their increased risk of restenosis after coronary intervention [18, 19]. Several mechanisms like the release of growth factors from activated platelets as well as the secretion of stimulatory mediators from inflammatory cells have been shown to induce SMC proliferation during atherogenesis and restenosis formation [9]. Conflicting data exist on the role of C-peptide in SMC proliferation. A recent report coming from Kobayashi et al. demonstrated an inhibition of rat SMC proliferation after 3 days of treatment with human C-peptide under high glucose concentrations [20]. These effects observed at high C-peptide concentrations (100 nmol/L) were mediated by an inhibition of PDGF-beta receptor expression as well as by a reduction in p42/p44 MAP-kinase phosphorylation. These data are in contrast to results from our own group demonstrating an induction of SMC proliferation upon C-peptide stimulation [16]. In our experimental setting, human C-peptide induced the proliferation of human SMCs while rat C-peptide exhibited similar effects in rat SMCs. These mitogenic effects of C-peptide on SMCs were inhibited by a specific inhibitor of Src-kinase as well as transfection of cells with short interference RNA (siRNA) against Src. Similarly, inhibition of PI3-kinase and MAP-kinase reduced C-peptide-induced SMC proliferation. Moreover, C-peptide induced Src phosphorylation as well as activation of PI3-kinase and ERK 1/2, promoting the concept that these signalling molecules are involved in C-peptide induced SMC proliferation. Moreover, C-peptide increased cyclin D1 expression as well as phosphorylation of the retinoblastoma protein (Rb). These data suggest that C-peptide induces SMC proliferation and demonstrate another mechanism how C-peptide may contribute to plaque development and restenosis formation in patients with insulin resistance and early type 2 diabetes. Different experimental conditions may account for the discrepant results between the two studies cited and future work is warranted to further elucidate this issue.

## 6. C-PEPTIDE AND MICROVASCULAR THROMBUS FORMATION IN MICE

Interesting experimental data demonstrated a delay of microvascular thrombus formation in normal and diabetic mice upon high-dose C-peptide treatment. Such mechanisms were most likely mediated by a reduction in endothelial plasminogen activator inhibitor 1 (PAI-1) expression in arterioles and venules, but not by an effect on platelet activation. Interestingly, concomitant treatment with insulin abolished the effect of C-peptide on microvascular thrombus formation [21]. These data suggest that C-peptide could exhibit antithrombotic actions in vivo.

Additional effects of C-peptide on endothelial function as well as on endothelial-leukocyte interaction are discussed by Kunt and Pfutzner in the same issue of this journal.

## 7. SUMMARY AND FUTURE PROSPECTS

The majority of data described above suggest that C-peptide may promote lesion development in patients with type 2 diabetes mellitus and insulin resistance, while the application of C-peptide in type 1 diabetic patients who lack C-peptide has been shown to improve diabetic microvascular complications such as diabetic neuropathy. Is the potential proatherogenic action of C-peptide in contrast to such clinical benefits of C-peptide treatment in patients with type 1 diabetes?—Definitely not. When one compares the situation of C-peptide in type 1 and type 2 diabetic patients with the clinical presentation of hypo- and hyperthyroidism, C-peptide's effects are not contradictory. L-thyroxine treatment in patients with hypothyroidism is without any doubt beneficial, but elevated levels of L-thyroxine in those with hyperthyroidism can cause serious clinical manifestations. Similar mechanisms may apply for C-peptide: supplementation of C-peptide in type 1 diabetic patients may be beneficial while an increase in C-peptide levels in patients with insulin resistance and type 2 diabetes may be harmful.

Further studies in animal models of arteriosclerosis are warranted to examine whether the hypothesis of C-peptide's proatherogenic effects holds true in vivo. Moreover, additional work is needed to identify the C-peptide receptor. Such a receptor could be an interesting therapeutical target for both, patients with type 1 or type 2 diabetes. Activating such a receptor could be beneficial in type 1 diabetic patients, while blocking of C-peptide receptors in patients with insulin resistance and early type 2 diabetes may help to prevent vascular complications from early on.
